# Preparation and Angle-Dependent Optical Properties of Brown Al/MnO_2_ Composite Pigments in Visible and Infrared Region

**DOI:** 10.1186/s11671-017-2035-7

**Published:** 2017-04-08

**Authors:** Yunfeng Liu, Jianliang Xie, Mei Luo, Bo Peng, Longjiang Deng

**Affiliations:** 1 National Engineering Research Center of Electromagnetic Radiation Control Materials, University of Electronic Science and Technology of China, Chengdu, 610054 People’s Republic of China; 2 State Key Laboratory of Electronic Thin Films and Integrated Devices, University of Electronic Science and Technology of China, Chengdu, 610054 People’s Republic of China

**Keywords:** Aluminum flake, Angle-dependent pigments, Manganese dioxide, Reflectance, Infrared emissivity, Lightness

## Abstract

Traditional low infrared emissivity coatings based on aluminum flakes cannot own low IR emissivity and low lightness simultaneously. Herein, a new simple efficient method for the synthesis of brown Al/MnO_2_ composite pigments with low IR emissivity and low lightness is reported, through forming MnO_2_ layer on aluminum flakes by thermal cracking, then altering the shape and forming nanoshell by stirring in hot flowing liquid. The results indicate that the MnO_2_ particles, which have tetragonal structure with high crystallinity, are needlelike and forming a complete shell on the aluminum flakes. The optical properties of composite pigments can be tuned by mass of KMnO_4_ added in precursor and time of hot flowing. Strong angle-dependent optical effects are observed in five different angles through multi-angle reflectance spectrum, while low lightness and low IR emissivity are preserved. This work is expected to provide a new route for the preparation of colored aluminum effect pigments in low infrared emissivity coatings.

## Background

Effect pigments which have the angle-dependent optical effect are widely used in many fields [1–4]. Many studies are focused on color pearlescent pigments which are based on mica [5–7]. As a matter of fact, aluminum flaky pigment of single particle [8] or in a multi-layer structure as substrate are becoming of increasing interest recently in decorative material, heat insulation coatings, and security applications, due to their low infrared (IR) emissivity and other special optical properties [9–11]. As we know, many methods have been developed to prepare chromatic aluminum flakes, such as oxidation [12], physical/chemical vapor deposition [13–15], and chemical liquid deposition [16, 17]. A kind of aluminum effect pigments which has one metal oxide layer consisting of iron, manganese, copper, vanadium, etc. and an enveloping organic polymer layer is produced by wet chemical oxidation method [18]. But the binding force between the layer and the substrate aluminum is not strong through this sol–gel process. A radical polymerizable resin layer have been coated on aluminum pigment and then adhering coloring pigments by ball milling to fabricate colorful aluminum pigments [19]. However this organic layer results in the drastic increase of infrared emissivity. Aluminum flakes have been decorated by oxidizing in a water-in-oil emulsion comprising a surfactant in the presence of a base [20]. However, the L* value is still up to 96 at 15° in CIELAB. In a word, all of these methods are complicated, device dependent, or unstable in coating quality. Meanwhile, the lightness, gloss, and visible (VIS) reflectance of these pigments are very high which are needed to be as lower as possible in practice [21].

An efficient way to avoid these problems is to introduce the thermal cracking—hot flowing method which is developed to prepare silver layer on silica spheres [22]. The advantages of this method are that the layer is smooth due to the surface tension and the thickness is controllable. In this paper, this method is applied to fabricate brown Al/MnO_2_ composite pigments with low lightness, low infrared emissivity, and angle-dependent effects. We systematically discuss the influence of reaction conditions, such as mass of KMnO_4_ (*M*_KMnO4_) added in precursor and time of hot flowing (*t*_hf_), on morphology, reflectance of variable angles, lightness, and colors of coatings.

## Methods

The aluminum flakes (radius = 23 μm) of 5 g, potassium permanganate (KMnO_4_) of 2.2 g and 4.4 g (*M*_KMnO4_), ethanol of 30 ml, and zirconia balls of 50 g were charged in milling container to form a uniform precursor. The mixing process was performed for 1 h under rotation speed of 250 rpm, then collected the mixtures by suction filtration and added them in a ceramic crucible. The crucible was put inside a thermostat set at the temperature of 340 °C (temperature of thermal cracking, *T*_tc_) for 24 h to format the Al/MnO_2_ mixtures. After cooling down, the mixtures of 0.5 g and paraffin oil of 100 ml were added in a three-mouth flask and stirred at 130 °C for 24 or 48 h (time of hot flowing, *t*_hf_). Finally, the products were centrifugalized and washed with n-heptane and deionized water. The details of samples are shown in Table 1.

**Table 1:** The composition, reaction condition of the samples

Samples	*M*_KMnO4_/g	*T*_tc_/°C	*t*_hf_/h
S0 (Al flake)	–	–	–
S1	2.2	340	24
S2	2.2	340	48
S3	4.4	340	24
S4	4.4	340	48

The samples were characterized by X-ray diffraction (XRD) (SHIMADZU, XRD-7000 with CuKa radiation) and field emission scanning electron microscopy (FE-SEM; JEOL JSM-7600 F). For optical characterization, paints containing Al/MnO_2_ composite pigments were prepared by mixing Al/MnO_2_ powder 20%, lacquers 60%, and thinner 20%. Then, the films were painted by these mixtures onto microslides. The VIS spectral reflectance and CIE (International Commission on Illumination) L*a*b* with different angles (15°, 25°, 45°, 75°, and 110°) were measured by the angle dependence spectrophotometer (X-Rite, MA98XRB, D65 illuminant). Total infrared reflectance spectrum (3–21 μm) was measured by a Fourier transform infrared spectrometer (BRUKER, Tensor27) with integrating sphere attachment (BRUKER, A562). Total visible and near infrared (VIS/NIR) reflection spectrum (380–2300 nm) was measured by UV/VIS/NIR spectrophotometer (Perkin–Elmer, Lambda 750).

## Results and Discussion

### XRD Patterns

**Fig. 1:**
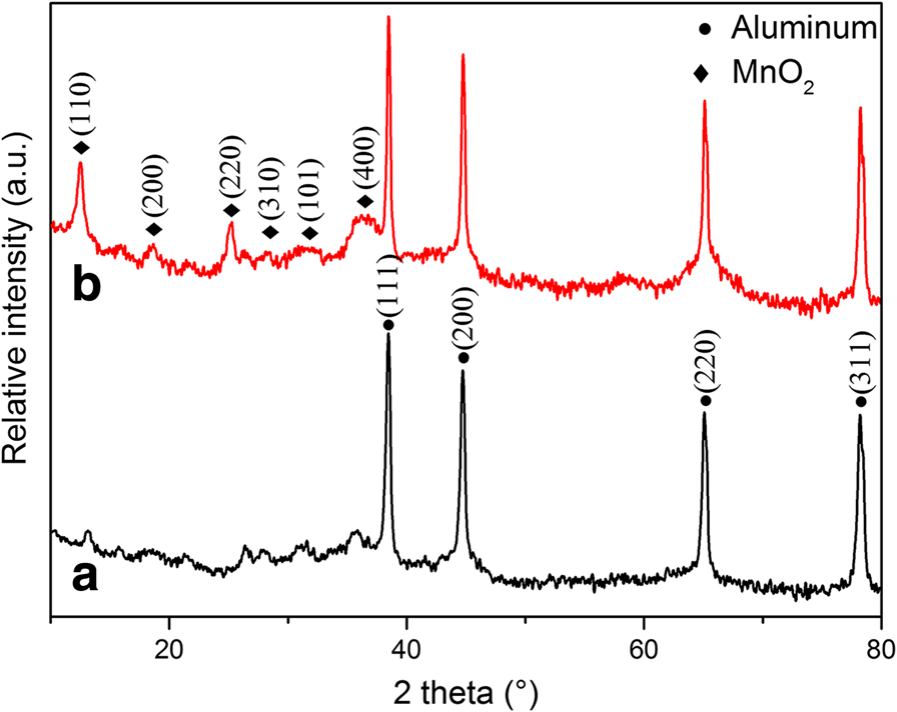
XRD analysis for Al/MnO_2_ composite pigments with (*a*) S2 and (*b*) S4

X-ray powder diffraction (XRD) of Al/MnO_2_ composite pigments are shown in Fig. 1. There are four strong diffraction peak of aluminum phase centered at 38.47°, 44.76°, 65.08°, and 78.25° in 2*θ* (JCPDS no. 85-1327). And the diffuse reflections centered at 2*θ* = 12.54°, 18.52°, 25.24°, 28.52°, 32.01°, and 36.14° which can be assigned to the (110), (200), (220), (310), (101), and (400) reflections of a tetragonal MnO_2_ phase (JCPDS no. 72-1982). The intensity of diffraction peaks of MnO_2_ of S4 is stronger than S2 owing to the higher *M*_KMnO4_ added in precursor.

### SEM Analysis

**Fig. 2:**
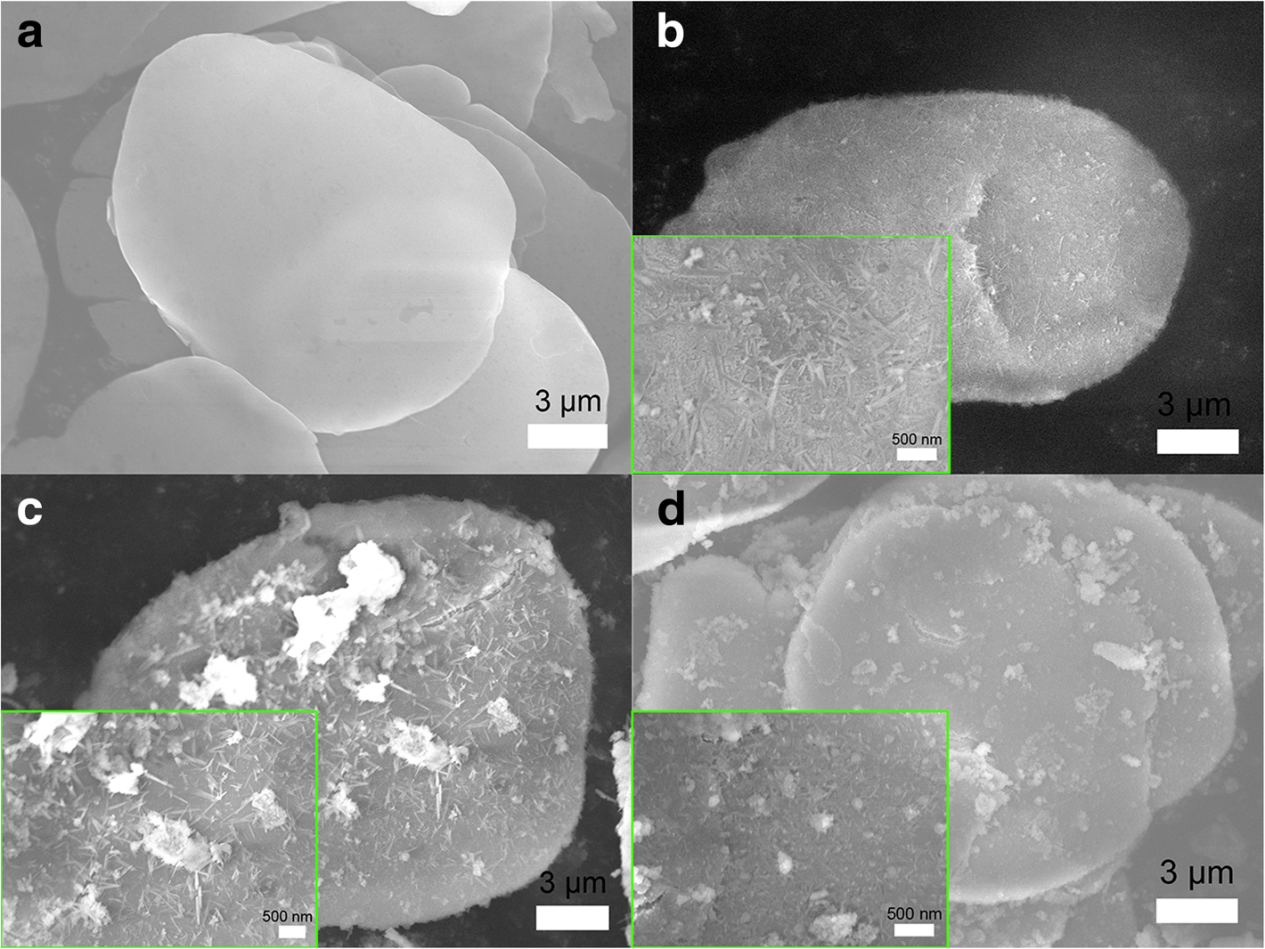
The SEM micrographs of the Al/MnO_2_ composite pigments with **a** aluminum flake, **b** S2, **c** S3, and **d** S4

The surface micrographs of Al/MnO_2_ composite pigments are shown in Fig. 2. From Fig. 2a, it can be seen that the surface of raw aluminum flake is smooth, flat, and mirrorlike. The Al/MnO_2_ composite pigment (S2) which is added in low *M*_KMnO4_ and after 48 h hot flowing is shown in Fig. 2b. The MnO_2_ particles form a complete shell on Al/MnO_2_ composite pigments. And in a larger scale, the needlelike MnO_2_ particles owing to the tetragonal crystal structure are well dispersed on the surface of aluminum flake. Figure 2c, d (S4) is the morphology of composite pigments with high *M*_KMnO4_, and the difference of these two samples is *t*_hf_ varying from 24 to 48 h. We can find that a MnO_2_ shell is prepared whereas the excess amount of MnO_2_ are agglomerated on the surface. These aggregation will help to reduce the visible light reflectance but result in a dramatic increase of heat accumulation caused by infrared absorption. Furthermore, longer *t*_hf_ will make the MnO_2_ particle small, and the MnO_2_ shell is more uniform compared in Fig. 2c and Fig. 2d. In fact, during the flowing process, the paraffin oil penetrates into the pores between MnO_2_ particles on the surface of aluminum flake which arise from the thermal decomposition of KMnO_4_. When stirring, the shear force between solid–liquid interfaces separates the MnO_2_ particles brings down the particle size and makes them distribute evenly. This process can be enhanced by extending *t*_hf_, thus tending to form a uniform coating of MnO_2_ on the surface of aluminum flake.

### Optical Properties

**Fig. 3:**
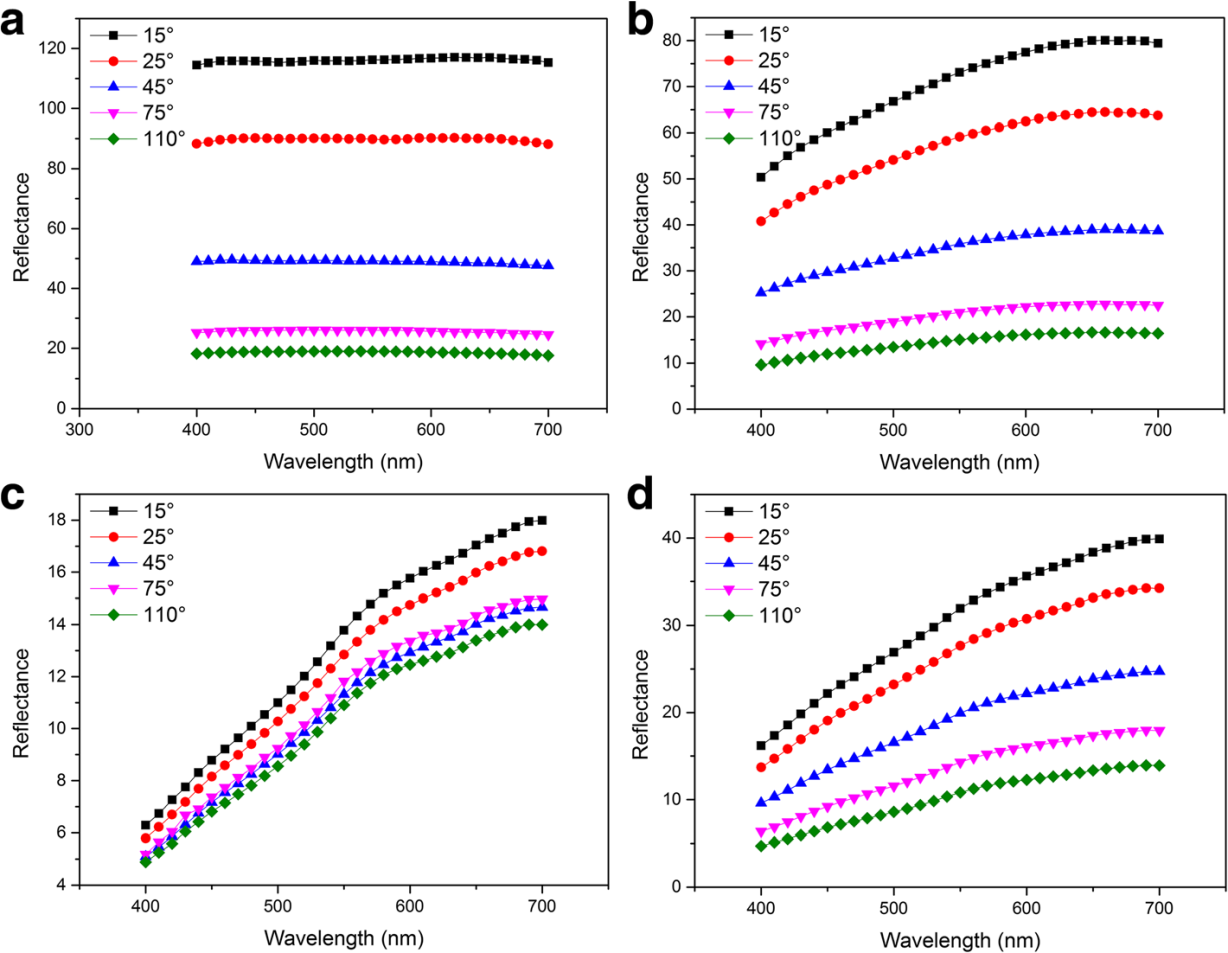
The visible reflectance spectra via variable angles of Al/MnO_2_ composite pigments with **a** aluminum flake, **b** S2, **c** S3, and **d** S4

The visible reflectance spectra with five different observation angles of aluminum flakes and Al/MnO_2_ composite pigments are shown in Fig. 3. The reflectance of raw aluminum flakes (Fig. 3a) is almost the same with increasing wavelength in all five angles. The value decreases while increasing the viewing angle and the reflectance reach the maximum of 116% at 15°. Figure 3b, d shows the noticeable effect of *M*_KMnO4_ on the multi-angle reflectance spectrum of Al/MnO_2_ composite pigments. All the value of reflectance arise with increasing wavelength in these curves, due to that MnO_2_ particles have the absorption peak centered at 400 nm [23]. Due to the lower *M*_KMnO4_ added in precursor, the reflectance of S2 (Fig. 3b) is higher than S4 (Fig. 3d) in all five angles, and the value of S2 increases sharply than S4 with the increasing angles. Moreover, the viable range of reflectance of S2 is from 9.6 to 80.1, which varies larger than S4 from 4.7 to 39.9. It indicates that S2 has stronger angle-dependent optical effects with five different angles from 15° to 110° than S4. Meanwhile, in *t*_hf_ of 24 h (Fig. 3c), the reflectance curves of different angles of S3 are very close and show weak angle-dependent effect. However, as shown in Fig. 3d, the effect of S4 becomes obvious by extending *t*_hf_ to 48 h. The results show that the angle-dependent effect of Al/MnO_2_ composite pigments is clearly affected by *t*_hf_.

**Fig. 4:**
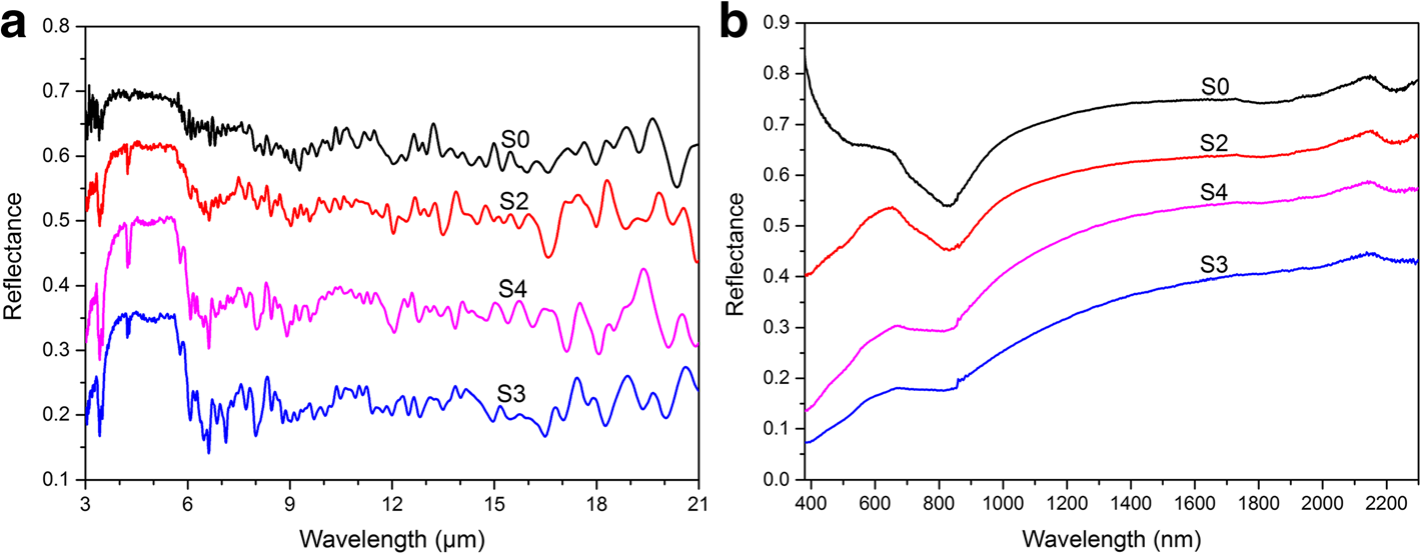
Total reflectance spectra of the Al/MnO_2_ composite pigments in **a** IR region 3–21 μm and **b** VIS-NIR region 380–2300 nm

The total reflection spectra of Al/MnO_2_ composite pigments in 3–21 μm and 380–2300 nm are shown in Fig. 4. Aluminum flake (S0) has the highest reflectance in both IR and VIS-NIR region, and *M*_KMnO4_ and *t*_hf_ have much effect on the total reflectance of composite pigments. In IR region (Fig. 4a), the reflectance of S4 is lower than that of S2 owing to high *M*_KMnO4_. Meanwhile, the reflectance of S4 is higher than that of S3, due to the smoother surface caused by longer *t*_hf_. Similar rules can be found in VIS-NIR region (Fig. 4b). According to the Kirchhoff’s law, the relationship between the IR emissivity (*ε*) and reflectance (*R*) of non-transparent material can be expressed as follows:

1 $$ \varepsilon =1\hbox{-} R $$

That means after being processed, S2 preserve low IR emissivity and low visible reflectance which is beneficial to reduce the light pollution. S4 has lower visible reflectance than S2, but the emissivity of S4 is lower than 0.5, which cannot be used in low IR emissivity coating. In summary, more *M*_KMnO4_ and less *t*_hf_ result in the lower total reflectance of composite pigments, and S2 is supposed to be a good choice of effect pigments in low IR emissivity coatings.

**Fig. 5:**
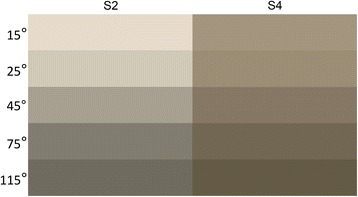
Visual effects of Al/MnO_2_ composite pigments (S2, S4) in different observed angles (D65 illuminant)

The visual effects of Al/MnO_2_ composite pigments in different observed angles are shown in Fig. 5. The colors of samples are light at 15° and become dark at larger angle. Owing to the low *M*_KMnO4_ in reaction, the color of S2 is lighter than S4. The CIE L*a*b* values with varying angles of Al/MnO_2_ composite pigments are shown in Table 2. The CIE L*a*b* color scale is a standard scale for comparison of color values so that color values can be easily compared. In CIE L*a*b* color space, L* is the lightness from perfect reflecting diffuser to pure black, a* is red to green, and b* is yellow to blue [24]. From Table 2, it can be seen that the lightness decreases with the growth of measuring angle. The lightness of S2, which changes widely, varies from 88.17 at 15° to 45.41 at 115°, compared with S4 from 62.95 at 15° to 38.90 at 115°. In the same angle, the L* value of S2 is higher than that of S4. The colors of Al/MnO_2_ composite pigments at all observed angles are brown, and the values of a* and b* change little. In general, it indicates that Al/MnO_2_ composite pigments have strong angle-dependent optical effects.

**Table 2:** The CIE L*a*b* values of Al/MnO_2_ composite pigments (S2, S4)

Sample	Angle	L*	a*	b*
S2	15°	88.17	1.42	10.78
	25°	81.05	1.13	9.86
	45°	66.13	1.07	8.20
	75°	52.59	0.87	7.46
	110°	45.41	1.09	7.63
S4	15°	62.95	2.65	14.92
	25°	59.22	2.42	14.50
	45°	51.40	2.19	13.61
	75°	44.25	2.27	13.39
	110°	38.90	2.45	12.85

## Conclusions

In conclusion, brown Al/MnO_2_ composite pigments are fabricated through coating MnO_2_ layer on aluminum flakes by a new thermal cracking and hot flowing method. The composite pigments are termed a brown metallic shade owing to the absorption of MnO_2_ shell. The variation of reflectance, lightness, and color of composite pigments are huge in different observed angles. When in low *M*_KMnO4_ and *t*_hf_ of 48 h, the Al/MnO_2_ composite pigments which have strong angle-dependent optical effects and low IR emissivity will supposed to be a good choice of effect pigments in low IR emissivity coatings.

## Abbreviations

*M*_KMnO4_: Mass of KMnO_4_
R: Reflectance
*t*_hf_: Time of hot flowing
*T*_tc_: Temperature of thermal cracking
E: Emissivity
